# Outcomes of Children and Adolescents Admitted with Diabetic Ketoacidosis at Kenyatta National Hospital (KNH), Kenya

**DOI:** 10.1155/2020/8987403

**Published:** 2020-10-20

**Authors:** Sophie Nyaombe Musoma, Anjumanara Omar, Beatrice Chepngeno Mutai, Paul Laigong

**Affiliations:** Department of Paediatrics and Child Health, University of Nairobi, Nairobi, Kenya

## Abstract

**Background:**

Diabetic ketoacidosis (DKA) is an acute, major, life-threatening complication that mainly occurs in patients with type 1 diabetes mellitus and is the foremost cause of death in these children. Overall mortality in children with DKA varies from 3.4% to 13.4% in developing countries. There is a need to understand outcomes among children with DKA in sub-Saharan African countries.

**Objective:**

To determine the death rate and clinical outcomes of children and adolescents aged 0-18 years managed for DKA at Kenyatta National Hospital (KNH). *Study Methods*. This was a retrospective study carried out among children aged 0–18 years admitted with DKA at KNH between February 2013 and February 2018. The study site was the central records department at KNH. The inclusion criteria were children aged 0-18 years admitted with a diagnosis of DKA based on the ISPAD guidelines biochemical criteria.

**Results:**

Out of the 159 files reviewed, the median age of children was 13 years (IQR 10-15). 41.1% of patients had severe DKA while 35.7% had moderate DKA. We reported a mortality of 6.9% while 93.1% of children recovered and were discharged home. The median duration of hospital stay was 8 days. High risk of mortality was reported among children who had high serum creatinine (OR 5.8 (95% CI 1.6-21.2)), decreased urine output (OR 9.0 (95% CI 2.2-37.3)), and altered level of consciousness (OR 5.2 (95% CI 1.1-25.1)).

**Conclusion:**

DKA-associated mortality in our study was low at 6.9%. High serum creatinine, decreased urine output, and altered level of consciousness were associated with a significantly higher risk of mortality.

## 1. Introduction

Diabetic ketoacidosis (DKA) has been recognized as the main complication that is a potentially fatal emergency in children and adolescents with type 1 diabetes mellitus. Early recognition and treatment in patients with new-onset diabetes are essential in the prevention of this life-threatening complication of diabetes [1]. DKA presents with a clinical triad of biochemical abnormalities that include hyperglycaemia (blood glucose level > 11 mmol/L or 200 mg/dL), venous pH of <7.3 or serum bicarbonate level < 15 mmol/L, and ketonemia (blood*β* − hydroxybutyrate ≥ 3 mmol/L) or ketonuria [2]. Patients, particularly children and teenagers, may present with ketoacidosis as the first manifestation of the disease [3]. Most children in sub-Saharan Africa present to health facilities with DKA at first diagnosis following a missed diagnosis of uncomplicated diabetes [4]. DKA is the most common cause of death in children with T1DM, and the most common complications of DKA are cerebral edema, impaired renal function, and secondary infections [5]. The mortality rate associated with DKA among children in industrialized countries has declined to 0.15%-0.31% [6, 7]. However, in less developed countries, the risk of death from DKA remains high at 3.4% to 13.4%, and children may die before receiving adequate treatment or during treatment [7–9]. Timely recognition of DKA and appropriate subsequent management are important to minimize complications and death. The objectives of this study were to determine the death rate and clinical outcomes of children and adolescents managed for DKA at KNH and describe the average length of stay in hospital and factors associated with mortality among this group of children.

## 2. Methods

Kenyatta National Hospital is the largest national referral facility in Kenya and is affiliated to the University Of Nairobi College Of Health Sciences as a teaching hospital. Children seen at this facility with DKA are mainly referrals from lower-level health facilities. Children aged 12 years and below are admitted to the paediatric wards while older children are admitted to medical wards.

### 2.1. Study Design

This was a retrospective observational study conducted over a 5-year period (February 2013-February 2018).

### 2.2. Study Population

The study population consisted of children aged 0-18 years admitted with DKA at KNH during the study period with a diagnosis of DKA based on the following biochemical criteria as per the ISPAD guidelines.

- Hyperglycemia (random blood glucose) >11 mmol/L (≈200 mg/dL)).
- Venous blood pH < 7.3 or bicarbonate < 15 mmol/L
- Ketonuria >2 Plus in urine samples

Children with a clinician's diagnosis of DKA but with missing data on some of the diagnostic parameters stated above were excluded.

### 2.3. Study Outcomes

- Proportion of children admitted with DKA who died
- Average length of hospital stay
- Factors associated with mortality among children with DKA

## 3. Results

We reviewed the medical records of 159 children admitted with DKA during the 5-year period preceding the study. All children who met the eligibility criteria during this period were recruited. The median age of children was 13 years (IQR 10-15), and 91 (57.2%) were females. Forty-nine (30.8%) children aged less than 12 years were admitted to the paediatric ward while 19 (12%) were admitted to the paediatric ICU.

Out of 68 admissions to the paediatric ward, only 16 (10%) were below 6 years of age. Seventy children (44%) aged twelve years and above were admitted to the medical wards, and 21 (13.2%) were admitted to the main intensive care unit (ICU).

Out of the 159 children, 103 (64.8%) had a previous diagnosis of diabetes (prior to the current admission) while 56 (35.2%) were newly diagnosed. Among those previously diagnosed, 88 (85.4%) were on mixtard insulin and 11 (10.7%) were on Lantus+short-acting insulin, and for 4 (2.5%) children, their records did not indicate the type of insulin they were on (Table 1).

Out of the 159 children admitted with DKA, 23 (41.1%) had severe DKA, 20 (35.7%) had moderate DKA, and 13 (23.2%) had mild DKA (Table 1). Only 6 out of 16 children aged ≤ 6 years had DKA severity documented. Of these, 1 (16.7%) had severe DKA, 4 (66.6%) had moderate DKA, and 1 (16.7%) had mild DKA.

### 3.1. Clinical Outcomes of Children Admitted with DKA

**Figure 1:**
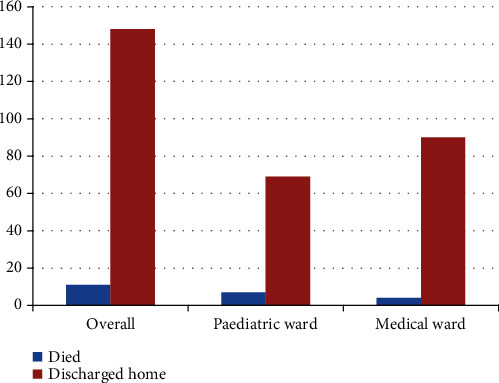
Clinical outcomes of children admitted with DKA.

Out of the 159 children admitted, 11 died giving a mortality rate of 6.9%. Six (3.8%) of the deaths occurred in the paediatric ward while 5 (3.1%) were from the medical ward (Figure 1).

### 3.2. Factors Associated with Mortality among Children and Adolescents with DKA

15% of the study population had high serum creatinine while 12% had reduced urine output at some point during the inpatient period. Seventy-six patients (47.8%) manifested some signs of altered level of consciousness during the same period while 88 (55.3%) of the children had temperature readings above 37.5 degrees Celsius at one point or another during the inpatient stay. Normal temperature readings were noted throughout the inpatient stay for 68 (42.8%) children, and 3 (1.9%) had no temperature readings documented.

High serum creatinine, low urine output, and altered consciousness were all associated with a higher risk of mortality. Children with high creatinine had a fivefold higher risk of mortality compared to those with normal creatinine (OR 5.8 (95% CI 1.6-21.2)), those with decreased urine output had a 9-fold higher risk (OR 9 (95% CI 2.2-37.3)), and children with altered consciousness were 5 times more likely to die compared to those who remained alert throughout the entire inpatient period (OR 5.2 (95% CI 1.1-25.1)). We found no association between mortality and age, sex, DKA severity, admission ward, type of insulin, missed insulin doses, or the presence of fever (Table 2).

### 3.3. Association between Age Less than 6 Years and Risk of Mortality

We reported only 1 death among children aged below 6 years and did not find a significant association between this age category and the risk of mortality (OR 1.13 (95% CI 0.13-9.43)) (Table 3).

### 3.4. Association between Time of Diabetes Onset and Risk of Mortality

We reported 8 (5%) deaths among children with previously diagnosed diabetes and only 1 (1.9%) death among children with newly diagnosed diabetes. Hitherto, we found no significant association between the time of diagnosis and mortality (OR 1.5 (95% CI 0.38-5.85)) (Table 4).

### 3.5. Duration of Hospital Stay

The overall median duration of hospital stay for children was 8 days (IQR 5-13). Children admitted to the paediatric and main hospital ICUs had a similar median stay of 5 days (IQRs 3-7 and 3-10, respectively). The median stay in the medical and pediatric wards was 10 (IQR 6-14) and 8 days (IQR 5-10), respectively. The summary data on the duration of hospital stay is presented in Table 5.

## 4. Discussion

In this study, out of 159 children admitted with DKA, we reported a mortality rate of 6.9%. This mortality is high compared to that reported in studies from developed countries that have reported mortality rates of 0.15%-0.31% among children admitted with DKA [6, 7]. The mortality rate in our study is however comparable to the mortality rates reported in studies conducted in developing countries where it varies from 3.4% to 13.4% [7–9]. Despite the fact that DKA severity was not documented for 9 of the deaths that we reported, we attributed the high mortality rate to the fact that KNH is a tertiary hospital and most of our study participants were referrals from lower-level facilities and already had severe DKA at the time of presentation. Information on the place of residence and use of alternative medicines prior to presentation was not obtained as it was not documented in many of the patients' records. Of note however is the fact that KNH serves a catchment population mainly from the low socioeconomic urban settlements within Nairobi and its environs with a small proportion derived from the middle class, and delay in seeking care is likely to have contributed to high mortality.

From our study results, we noted that children with high serum creatinine were 5 times more likely to die while those with anuria were 9 times more likely to die. Renal failure has been largely recognized to be an independent risk factor for mortality in DKA in studies conducted in India and other parts of the world [9, 10]. Intrinsic renal failure was reported to have occurred in 11.5% of children with DKA in South India, with associated case fatality rates of 40% to 72% [11]. In DKA, high blood glucose levels lead to increased urination and volume depletion predisposing children to acute kidney injury. Fluid restriction in a child with sepsis and hypovolemic or septic shock for fear of cerebral edema during the management of DKA may cause renal failure. AKI in our study may have resulted from poor fluid management during the initial stages of DKA. Although the ISPAD protocol guidelines for DKA management are available within both the medical and paediatric wards at KNH and healthcare workers are trained on their use, poor adherence to these guidelines by healthcare workers has been noted.

Altered consciousness was independently associated with a fivefold higher risk for mortality in our study (OR 5 (95% CI 1.1-25.1)). Altered consciousness is a common presentation of cerebral edema which has been associated with approximately 43% mortality in children with DKA in developing countries [7, 12]. In this study, although we were not able to report on other clinical signs of cerebral edema in children due to poor documentation, cerebral edema has been reported in studies to be the leading cause of altered consciousness among children with DKA. In a cohort of children admitted at a pediatric intensive care unit in north India and another study in Chennai, India, cerebral edema occurred in 26% and 24% of the study population, respectively [13, 14]. Out of 76 patients who had altered consciousness, 11.8% died which is a lower proportion compared to that reported from other developing countries [7].

We noted a lower mortality rate of 3.1% among older children and adolescents admitted to the medical wards compared to 3.8% among younger children admitted to the paediatric ward; however, this difference in mortality was not statistically significant. Although mortality in children younger than 6 years was low at 0.6%, we had only 16 children within this age category with 1 death reported. A study done by Kao et al. on the incidence and trends of DKA in children and adolescents with type 1 diabetes in British Columbia, Canada, reported that younger age at diagnosis (<5 years) was associated with a greater risk of DKA at the time of diabetes diagnosis [15]. We also did not report any mortality in the ICU which we attributed to close monitoring of patients in the ICU and with a prompt institution of appropriate interventions in the event of clinical deterioration.

Temperature above 37.5 degrees, an indicator of possible infection, was not independently associated with mortality in our study. This finding contradicts what was reported in the study conducted in Chennai, India, where infection was identified as a significant risk factor and a contributor to poor outcomes in children with DKA [16]. We reported severe DKA in 41.1% of children which is higher compared to the study done in Italy by Rabbone et al. where severe DKA was reported in 15.3% of children aged <18 years [17]. It was also higher than what was reported in another study by Cherubini et al. on DKA frequency at diagnosis of type 1 diabetes mellitus (T1DM) among children aged < 15 years. Cherubini reported DKA in 40.3% of children at diagnosis of TIDM and severe DKA in 11.2% of children [18]. The study by Rabbone reported severe DKA in 16.7% of the children aged < 6 years, and we reported a similar proportion of children with severe DKA in this age group [17]. Contrary to the findings from previous studies, we did not find any association between DKA severity, age, sex, type of insulin, missed insulin doses, and mortality, and this could be explained by the fact that the examination for mortality-associated factors was a secondary objective in our study and our sample size may not be adequate to assess for this. This is confirmed by the wide confidence intervals that we reported for most of the associations.

We sought to determine the length of hospital stay (LOS) as a proxy indicator of the quality of DKA management offered at KNH. The median duration of hospital stay was 8 days and was similar at 8 (IQR 6-14) and 10 [5–10] days for the pediatric and medical wards, respectively. The duration of hospital stay in our study is comparable to that reported in a chart review of 151 patients admitted with DKA in Ethiopia by Desse et al. where nearly half of the patients (47%) had a long hospital stay of >7 days [19]. The LOS in our study was however much higher than the acceptable LOS of 3.4 days reported in studies in the US associated with the provision of quality care [20]. Longer hospital stay among DKA patients in the study by Desse et al. was related to poor DKA management setup, DKA management protocol, and patient characteristics [19]. We could not however in our study ascertain whether the long duration of hospital stay was purely associated with the quality of care or may have been due to other factors such as DKA severity or financial limitations.

## 5. Conclusions

DKA-associated mortality in our study was high at 6.9%. Increased serum creatinine, decreased urine output, and altered level of consciousness were significantly associated with a high mortality in our setup. We reported a long duration of hospital stay among children admitted with DKA with a median stay of 7 days.

The key to achieve a good outcome and prevent mortality is the prevention of DKA itself through early recognition, through support and supervision, and through proper blood glucose management in those with established diabetes. It is important that healthcare workers treating children and adolescents with diabetes and DKA follow the guidelines in the management of DKA.

## Figures and Tables

**Table 1:** Baseline characteristics of children admitted with DKA.

Patient characteristics	Frequency *N* (%)
Median age (IQR) in years	13 (10-15)
Age categories	
≤6 years	16 (10.1)
>6 years	143 (89.9)
Sex	
Male	68 (42.8)
Female	91 (57.2)
Admission ward (*n* = 159)	
Paediatrics	49 (30.8)
Medical	70 (44)
Paediatric ICU	19 (12)
Main ICU	21(13.2)
Time of diabetes diagnosis	
Previously diagnosed	103 (64.8)
Newly diagnosed	56 (35.2)
Type of insulin treatment (previously diagnosed *n* = 103)	
Mixtard	88(85.4)
Lantus+short-acting insulin	11 (10.7)
Missing record	4 (3.9)
Missed insulin (*n* = 103)	
Yes	27 (26.2)
No	76 (73.8)
Distribution based on DKA severity (*n* = 56)	
Mild	13 (23.2)
Moderate	20 (35.7)
Severe	23 (41.1)
Distribution of DKA severity for children aged ≤ 6 years (*n* = 6)	
Mild	1 (16.7)
Moderate	4 (66.6)
Severe	1 (16.7)

**Table 2:** Factors associated with mortality among children and adolescents with DKA.

Variables	Category (*n*)	Died, *n* (%)	Recovered, *n* (%)	OR (95% CI)	*p* value
Age in years	>12 (90)	4 (4.4)	86 (95.6)	0.41 (0.12-1.47)	0.211
	≤12 (69)	7 (10.1)	62 (89.9)	Ref	
Sex	Female (91)	8 (8.8)	83 (91.2)	2.09 (0.53-8.19)	0.355
	Male (68)	3 (4.4)	65 (95.6)		
Type of insulin the child is on	Mixtard (88)	7 (8.0)	81 (92.0)	0.86 (0.01-7.77)	1.0
	Lantus+short-acting insulin (11)	1 (9.1)	10 (90.9)	Ref	
Missed insulin doses	Yes (27)	2 (7.4)	25 (92.6)	0.920 (0.17-4.86)	1.0
	No (76)	6 (7.9)	70 (92.1)	Ref	
Serum creatinine levels raised >2 times above normal level	Yes (22)	5 (22.7)	17 (77.3)	*5.83 (1.60-21.2)*	*0.012*
	No (125)	6 (4.8)	119 (95.2)	*Ref*	
Anuria or reduced urine output of less than 0.5 mL/kg/hr for 12 hours	Yes (12)	4 (33.3)	8 (66.7)	*9.0 (2.17-37.3)*	*0.007*
	No (133)	7 (5.3)	126 (94.7)	*Ref*	
Level of consciousness	V/P/U (Abnormal) (76)	9 (11.8)	67 (88.2)	*5.24 (1.09-25.1)*	*0.029*
	A (normal) (80)	2 (2.5)	78 (97.5)	*Ref*	
Temperature above 37.5 degrees Celsius	Yes (88)	7 (8.0)	81 (92.0)	1.383 (0.39-4.93)	0.757
	No (68)	4 (5.9)	64 (94.1)	Ref	
DKA severity	Mild (13)	0 (0)	13 (100)	Ref	
	Moderate (20)	0 (0)	20 (100)	-	-
	Severe (23)	2 (8.7)	21 (91.3)	1.095 (0.965-1.242)	0.525
Admission ward (*n* = 159)	Paediatrics (49)	43 (87.8)	6 (12.2)	1.67 (0.49-5.70)	0.531
	Medical (70)	65 (92.9)	5 (7.1)	0.41 (0.12-1.46)	0.210
	Paediatric ICU (19)	19 (100)	0 (0)	-	-
	Main ICU (21)	21 (100)	0 (0)	-	-

**Table 3:** Association between age less than 6 and risk of mortality.

Variable				
Category (*n*)	Died *n* (%)	Recovered *n* (%)	OR (95% CI)	*p* value
≤6 years [16]	1	15	Ref	
>6 years (143)	10	133	1.13 (0.13-9.43)	1.0

**Table 4:** Association between the time of diabetes onset and risk of mortality.

Variable	Category (*n*)	Mortality, *n* (%)	Recovered, *n* (%)	OR (95% CI)	*p* value
Time of diabetes diagnosis	Previously diagnosed (103)	8 (5)	95 (59.8)	1.5 (0.38-5.85)	0.75
	Newly diagnosed (56)	3 (1.9)	53(33.3)	Ref	

**Table 5:** Duration of hospital stay.

Ward	Median length of hospital stay	IQR
Paediatric ward	10	6-14
Medical ward	8	5-10
Paediatric ICU	5	3-7
Main ICU	5	3-10
Overall duration of hospital stay	8	5-13

## Data Availability

The data will be available on request.
